# Rotating Hinge Knee Versus Constrained Condylar Knee Revision: A Single Centre, Retrospective Comparative Study

**DOI:** 10.7759/cureus.112443

**Published:** 2026-07-11

**Authors:** Habeeb Bishi, Irrum Afzal, Chao Wang, John Stammers, Suroosh Madanipour, Sarkhell Radha, Richard Field, Philip Mitchell, Sulaiman Alazzawi

**Affiliations:** 1 Trauma and Orthopaedics, The Shrewsbury and Telford Hospital NHS Trust, Shrewsbury, GBR; 2 Trauma and Orthopaedics, South West London Elective Orthopaedic, London, GBR; 3 Maths and Statistics, Kingston University London, London, GBR; 4 Trauma and Orthopaedics, The Royal London Hospital, London, GBR; 5 Trauma and Orthopaedics, St George's University Hospitals, London, GBR; 6 Orthopaedics, Croydon University Hospital, Croydon, GBR; 7 Orthopaedics, South West London Elective Orthopaedic Centre, Epsom, GBR

**Keywords:** constrained condylar implants, oxford knee score, patient reported outcome measures, revision total knee arthroplasty, rotating hinge implants

## Abstract

Introduction

In revision total knee arthroplasty, rotating hinge implants (RHK) have been presumed to result in higher complication rates and lower survivorship when compared to constrained condylar implants (CCK). This study aimed to compare patient-reported outcome measures (PROMs), complication rates and survivorship of RHK and CCK used in revision arthroplasty at a single, high-volume elective orthopaedic centre with a previously validated bespoke database.

Methods

Patients (n=108) who underwent revision knee arthroplasty with either CCK or RHK and matched our inclusion criteria were identified. EuroQol Five Dimensions (EQ5D), EuroQol Five Dimensions (EQ5D)-Health State and Oxford Knee Scores were collected pre-operatively and at one year post-operatively. Complication data was collected at six weeks, six months and one year post-operatively. National Joint Registry (NJR) data were interrogated, in addition to our orthopaedic database, to investigate implant survival with a maximum follow-up of 12 years.

Results

There was no statistical significant difference between RHK and CCK in implant survival at two to 12 years of follow up. In addition, we observed no statistical significant difference in the PROMs scores and complication rates of the two implants.

Conclusion

This study shows that both the RHK and CCK remain viable options in revision arthroplasty; the implant survival and complication rates were comparable. We recommend future research through prospective randomised control trials with long-term follow up to further investigate the use of CCK and RHK implants in revision knee arthroplasty.

## Introduction

Revision total knee arthroplasty (TKA) complicated by bone loss and ligament instability is typically more difficult than primary TKA and is linked with increased complication rates. As with primary TKA, revision knee arthroplasty aims to achieve sufficient stability with the least possible constraint. The most common implant used in revision knee arthroplasties is a posterior stabilised implant [1]. However, when this option does not provide adequate stability, a constrained condylar knee (CCK) or a rotating hinge knee (RHK) can be used to correct varus and valgus global ligamentous instability. The required constraint will also depend on the severity of bone loss and/or the functional integrity of the collateral ligaments [2]. CCK is most commonly used in comparison to the RHK on the assumption they have better longevity [3] and leaves the most constrained option (RHK) as a salvage option. However, this assumption is always debatable as many variables determine the outcome, including surgical technique, complexity of surgery and design of the prosthesis [3].

The earliest hinge knee prosthesis was designed with a fixed hinge and was associated with unacceptably high failure rates. RHKs were introduced to reduce the likelihood of the prosthesis loosening by decreasing the stress transferred at the bone-cement interface [3].

In general, RHK is a more constrained prosthesis and despite improvements made to their design, RHKs are still considered to have higher complication rates and lower survivorship when compared to CCK [4-6].

The primary aim of this retrospective study was to compare survivorship of RHK versus CCK in revision arthroplasty performed at a single, high volume elective orthopaedic centre. The secondary aim was to compare the clinical outcomes, including patient-reported outcome measures (PROMs) and complication rates between the two different prostheses. This article was previously presented as a meeting poster at the British Association for Surgery of the Knee Annual Congress in 2022 at South Wales, UK.

## Materials and methods

Patients for this study were identified from a prospectively compiled arthroplasty database held at a high-volume elective orthopaedic centre. Between 2008 and 2020, we identified 735 patients who underwent revision knee arthroplasty at the centre by multiple surgeons using different brands for CCK and RHK surgery. All the CCK implants were uncemented with or without a sleeve, depending on the individual case. All the RHK implants were cemented stems without the use of a cone. Patients with stage one revision for infection, revision of partial joint arthroplasty (unicompartmental or patellofemoral joint arthroplasty) to TKA, patella resurfacing following a primary TKA and a revision where a less constrained prosthesis mechanism was used were excluded. All other indications for revision were included. There were no other inclusion or exclusion criteria.

Of the 735 patients, we identified 108 patients (108 knees) who underwent revision knee arthroplasty with either CCK (63 cases) or RHK (45 cases) and matched our inclusion criteria.

There were 53 male patients and 55 female patients with a mean age at the time of operation of 72.4 (range 52 to 90) years. The indications for revision were listed in Table 1.

**Table 1:** Indications for revision TKA: Total knee arthroplasty; CCK: constrained condylar knee; RHK: rotating hinge knee.

Indication for revision	Number (n)	CCK (n)	RHK (n)
Aseptic loosening	45	25	20
Infection	20	15	5
Instability	14	3	11
Wear of polyethylene	9	9	0
Reason not recorded	8	6	2
Pain	5	4	1
Recurrent patella dislocation/subluxation	2	0	2
Stiffness	2	1	1
Mal-alignment of TKA	2	0	2
Peri-prosthetic fracture	1	0	1

A comprehensive dataset was collected, this included: pre-operative demographic data collection, pre- and post-operative PROMs and post-operative patient reported complications. The pre-operative data included age at the time of the operation, gender and body mass index (BMI). The PROMs data included pre-operative and one-year post-operative EuroQol-5 Dimensions (EQ-5D), EQ-5D Health State and Oxford Knee Score. Post-operative complication data collection included the incidences of dislocation, joint infection and peri-prosthetic fracture at six weeks, six months and one year post-operatively [4,5,6].

The National Joint Registry (NJR) consultant-level data up to March 2022 was reviewed to identify if any cases had undergone further revisions outside our region and were not captured by our database. The Kaplan-Meier survival analysis is based on this dataset. Any available post-operative imaging was also reviewed to identify if the same implants had been retained. 

Statistical analysis

A series of linear regression models were used to assess the difference between RHK and CCK for various outcome scores, including EQ-5D, Health State and Oxford Knee Score. Potential confounding factors such as age, BMI and gender were adjusted as covariates. Potential non-linear effects of the continuous variables (age and BMI) were investigated using fractional polynomials. In addition, unobserved heterogeneity was modelled as a latent class variable. The optimal number of classes was determined by Akaike information criterion.

Given the small number of events (re-revision) observed, the Fisher’s exact test was used to compare re-revision rates between RHK and CCK. All statistical analyses were performed using Stata Version 16 (StataCorp LLC, College Station, Texas, USA).

Ethical considerations

There was no additional patient contact and as such this project was performed as a service evaluation without the need for formal ethical approval.

## Results

Implant survival rate

**Figure 1:**
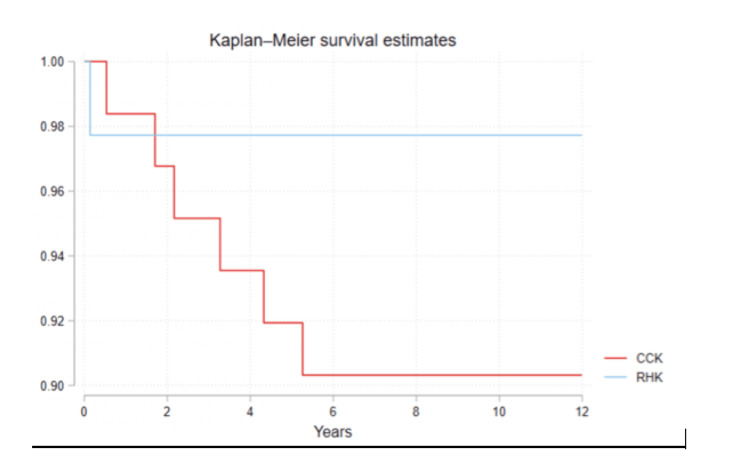
Kaplan-Meier survival graph in number of years CCK: constrained condylar knee; RHK: rotating hinge knee.

Of the 45 RHK patients, two (4.44%) had further revisions compared to seven (11.11%) from the 63 CCK patients. However, this does not represent a statistically significant difference (p=0.3) at a maximum follow-up time of 12 years (Figure 1).

Outcome scores

The preoperative outcome scores, including EQ5D, EQ-5D Health state and Oxford Knee Score, were comparable between the two groups of the patients with no statistical difference. This continued to be the case post-operatively at any time point of the follow-up (Table 2).

**Table 2:** Pre-operative and one-year post-operative outcome scores The effects of rotating hinge knees (RHK) relative to constrained condylar knees (CCK) on outcome scores, adjusted for age, BMI and gender. EQ5D: EuroQol 5-Dimension.

Time-interval	Mean outcome scores (SD) – RHK	Mean outcome scores (SD) – CCK	RHK relative to CCK with linear regression model	95% Confidence Interval
Pre-op EQ5D	0.25 (0.33)	0.33 (0.30)	-0.02	(-0.06,0.03)
1 year EQ5D	0.48 (0.37)	0.59 (0.33)	-1.5	(-4.20,1.19)
Pre-op Oxford	15.42 (8.25)	18.03 (9.07)	-4.89	(-13.11,3.33)
1 year Oxford	25.67 (11.34)	28.27 (11.68)	-0.02	(-0.09,0.04)
Pre-op Health State	57.87 (22.06)	65.67 (20.96)	-2.77	(-6.13,0.59)
1 year Health State	62.80 (23.90)	69.14 (19.44)	-4.83	(-10.44,0.78)

The complication rates were relatively low in both groups, but it is notable that patella subluxation was only noted in the RHK group (Table 3).

**Table 3:** Major post-operative surgical complications RHK: Rotating hinge knee; CCK: Constrained condylar knee.

Time interval	Joint infection		Peri-prosthetic fracture		Patella dislocation	
	RHK (n=45)	CCK (n=63)	RHK (n=45)	CCK (n=63)	RHK (n=45)	CCK (n=63)
6 weeks	2.22% (n=1)	0.00%	0.00%	0.00%	0.00%	0.00%
6 months	0.00%	0.00%	4.44% (n=2)	3.17% (n=2)	0.00%	0.00%
1 year	0.00%	1.59% (n=1)	0.00%	0.00%	2.22% (n=1)	0.00%

## Discussion

The results of our multi-surgeon study showed that there was no statistically significant difference in the survivorship of RHK revisions when compared to CCK revisions. There was also no significant difference in the functional outcome scores or the complication rates of the two implants.

Of the 735 revision knee replacements included in our selection criteria, a constrained implant design was used in 108 cases. Of the 108 cases, an indication for revision was instability, dislocation/subluxation, or malalignment of peri-prosthetic fracture in only 19 cases (17.59%). In this regard, the indication for revision reported to the NJR does not appear to provide any indication of the likelihood that a constrained device would be required. Further work is required to investigate which pre-operative factors best predict when a constrained implant will be needed.

One of the reasons why surgeons may be hesitant to use a RHK is the perception that the shortcomings of fixed hinge knees [6] will apply to the RHK designs. Early hinged knees such as the GUEPAR Offset hinge prosthesis were introduced in the 1970s [7]. These rigid hinge joints did not allow flexibility for medial rotation of the joint. The design features used in the early models of the hinge prostheses resulted in high stresses across the implant-cement-bone interfaces, excessive particulate wear debris and ultimately significantly high rates of complications and implant failure [8].

Reports of higher complication rates and poorer outcomes for RHK revisions may be attributed to selection bias, particularly if patients who received RHK revisions had greater pre-operative bone and soft tissue loss, with more ligamentous instability than patients selected for CCK [9].

Hermans et al. [10] compared the functional outcome scores between 18 patients with CCK and 22 patients with RHK. In their series, the indication for revision surgery was stiffness. The RHK group demonstrated significantly better post-operative knee functional outcome scores at the two-year follow-up in comparison to the CCK group, knee function improvement, knee pain improvement, greater maximal flexion, better maximal extension, greater flexion gain, and greater extension gain. Their results are different from our experience in this study, which may be due to the choice of the outcome score or the bias from patient selection i.e. only patients with idiopathic post-operative primary TKA arthrofibrosis were included in Herman et al. [10] as opposed to a range of pathologies in our own study.

A meta-analysis of 12 studies conducted by Yoon et al. [5] found that the proportion of knees in which short-term and medium-term survival rates were evaluated did not differ significantly between RHK and CCK prostheses. 

Five studies were assigned to the short-term (<five years) subgroup and three studies to the midterm (5-10 years) subgroup. For the short-term subgroup, the RHK group had a higher survival rate than the CCK group, but this difference was not statistically significant. For the midterm subgroup, the RHK group had a lower survival rate than the CCK group, but this difference was again not statistically significant.

Like the meta-analysis above, our series of patients, we found that there was no significant difference between the RHK and CCK with regards to survival rate (RHK 43/45; CCK 56/63; p=0.3) in our series of patients over a 12-year time period. As the understanding of the biomechanical effects of prostheses such as the RHK has improved, so have the survival rates of the modern RHK, with one paper quoting long-term survival rates of about 80% at 10 years [11]. This improved understanding would have contributed to the parity observed in this study between the RHK and CCK knee, in terms of survivorship.

Barnoud et al. [12] found that, when comparing outcomes of RHK and CKK implants used for first revision of TKA after mechanical failure, there were significantly better clinical and functional outcomes for who underwent revision using CCK prostheses when compared to RHK prostheses, regardless of indication (Knee Society Score or KSS-knee 70.5 (CCK) versus 60.7 (RHK) (p<0.003) and KSS-function 74.9 (CCK) versus 47.7 (RHK) (p<0.004) at 3.7 (2.0-9.4) years of mean follow-up. Our study found that there was no significant difference at one year between the RHK and CCK with regards to their effect on the measured outcome scores (EQ5D, Oxford Knee Score, and Health State Score). The reason for this difference may be that Barnoud et al. [12] only included cases where mechanical failure was the indication for revision whereas, in our study, there was no selection bias. Also cases with infection and aseptic loosening amongst others, as indications for revision, were included. 

Shen et al. [13] found that, in septic revision patients, the hinged knee prostheses had a significantly higher mean improvement in KSS, knee function score and modified KSS than the unlinked constrained group. Our study found that there was no significant difference in outcome scores between the CCK and RHK implant at one-year post operative follow-up. But again, this may be because we considered a range of indications for revision as opposed to only sepsis.

The perception that RHK has poor survivorship and higher complication rates may be based on studies and experiences of early hinged knee prostheses. Luttjeboer et al. [14] compared the use of RHK and CCK in revision TKA and found that there was a high relative number of conservatively treated complications in the CCK group compared to the RHK group but this number was not statistically significant (p=0.45). The CCK group also had a high relative number of reoperations (n=14) compared to none in the RHK group, with the number found to be statistically significant (p=0.018) Our study found that rates of major postoperative surgical complications were comparable between the two implants up to two years after the operation with no statistically significant difference found between the two. The reason for the difference in our experiences may be that Luttjeboer et al. only considered knees where instability was the indication for revision. RHKs have been shown to provide significant improvement in both pain and function scores after TKA revision for global instability [14,15].

There are limitations to the current study including its retrospective nature. A further limitation was that this study only assessed one year post-operative outcome scores which may not be reflected with longer term follow-up. Future work would include doing a long-term retrospective study.

## Conclusions

In conclusion, both RHK and CCK are useful options in revision arthroplasty and are viable in selected cases, but implant choice should remain individualised according to instability, bone loss, soft-tissue condition, and surgeon judgment. At our centre, constrained implant designs are being used in approximately 15% of all revision procedures. As such, the number of such operations will be relatively low in most centres and a multicentre, prospective, randomised trial with long-term (>10 years) follow-up may be helpful to best understand the best use of CCK and RHK implants in revision knee arthroplasty. In the absence of such data, surgeons should rely on their clinical assessment and careful pre-operative planning to determine implant selection on a case-by-case basis.
